# Minigene splicing assays reveal new insights into exonic variants of the *SLC12A3* gene in Gitelman syndrome

**DOI:** 10.1002/mgg3.2128

**Published:** 2023-01-03

**Authors:** Xiaomeng Shi, Hong Wang, Ruixiao Zhang, Zhiying Liu, Wencong Guo, Sai Wang, Xuyan Liu, Yanhua Lang, Irene Bottillo, Bingzi Dong, Leping Shao

**Affiliations:** ^1^ Department of Nephrology the Affiliated Qingdao Municipal Hospital of Qingdao University Qingdao China; ^2^ Department of Nephrology Qingdao Eighth People's Hospital Qingdao China; ^3^ Department of Dermatology Peking University First Hospital Beijing China; ^4^ Division of Medical Genetics, Department of Molecular Medicine Sapienza University, San Camillo‐Forlanini Hospital Rome Italy; ^5^ Department of Endocrinology the Affiliated Hospital of Qingdao University Qingdao China

**Keywords:** exon splicing, exonic variant, Gitelman syndrome, minigene assay, *SLC12A3*

## Abstract

**Background:**

Gitelman syndrome (GS) is a type of salt‐losing tubular disease, most of which is caused by *SLC12A3* gene variants, and missense variants account for the majority. Recently, the phenomenon of exon skipping, in which variants disrupt normal pre‐mRNA splicing, has been related to a variety of diseases. Therefore, we hypothesize that a certain proportion of *SLC12A3* variants can result in disease via interfering with the normal splicing process.

**Methods:**

We analyzed 342 previously presumed *SLC12A3* missense variants using bioinformatics programs and identified candidate variants that may alter the splicing of pre‐mRNA through minigene assays.

**Results:**

Our study revealed that, among ten candidate variants, six variants (c.602G>A, c.602G>T, c.1667C>T, c.1925G>A, c.2548G>C, and c.2549G>C) led to complete or incomplete exon skipping by affecting exonic splicing regulatory elements and/or disturbing canonical splice sites.

**Conclusion:**

It is worth mentioning that this is the largest study on pre‐mRNA splicing of *SLC12A3* exonic variants. In addition, our study emphasizes the importance of detecting splicing function at the mRNA level in GS and indicates that minigene analysis is a valuable tool for splicing functional assays of variants in vitro.

## Graphical abstract

The purpose of this study was to identify the effect of previously presumed missense SLC12A3 variants on pre‐mRNA splicing using bioinformatics tools and minigenes. The results revealed that, among ten candidate variants, six variants (c.602G>A, c.602G>T, c.1667C>T, c.1925G>A, c.2548G>C, and c.2549G>C) led to complete or incomplete exon skipping by affecting exonic splicing regulatory elements and/or disturbing canonical splice sites. It is worth mentioning that this is the largest study on pre‐mRNA splicing of SLC12A3 exonic variants, and our study emphasizes the importance of detecting splicing function at the mRNA level in GS and indicates that minigene analysis is a valuable tool for splicing functional assays of variants in vitro.

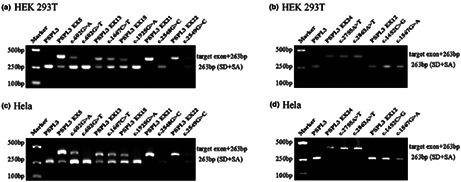

## 1 INTRODUCTION

Gitelman syndrome (GS) is a salt‐losing tubular disorder with autosomal inheritance, characterized by hypokalemic metabolic alkalosis in conjunction with significant hypomagnesemia and hypocalciuria (Parmar et al., 2021; Simon et al., 1996; Vargas‐Poussou et al., 2011). The vast majority of GS phenotype is related to inactivating variants in the *SLC12A3* gene (OMIM 600968), which is located on chromosome 16q13 with a total of 26 exons and encodes the thiazide‐sensitive NaCl cotransporter (NCCT) in the distal convoluted tubules of the nephron (Takeuchi et al., 2015; Vargas‐Poussou et al., 2011). Mutations in the *SLC12A3* gene can lead to the malfunctioning of NCCT, which in turn, disrupt the absorption of sodium and chloride ions from the tubular lumen (Parmar et al., 2021).

With the development and application of high‐throughput sequencing technology based on large‐scale parallel sequencing, an expanding list of rare variations about specific genetic diseases has been discovered in the clinic. To date, a total of 492 GS‐related gene variants have been documented in the Human Gene Mutation Database (HGMD, Professional 2020.10), including missense/nonsense variants (309, 63%), splicing (69, 14%), small deletions (56, 11%), small insertions (26, 5%), small indels (5, 1%), gross deletions (22, 4%), gross insertions/duplications (4, 1%), and complex rearrangements (1, 1%). The conclusion that transcripts from at least 74% of all multiexon genes were alternatively spliced has been drawn by using exon‐exon junction microarrays to analyze lots of RNA samples in a study (Johnson et al., 2003). Evidently, the relatively large number of exons (26) that encode NCCT suggests the existence of alternative splicing for *SLC12A3*.

Precursor messenger RNAs (Pre‐mRNAs) splicing, removing non‐coding insertion segments called introns and joining exons composed of protein‐coding regions together with two terminal untranslated regions, is an important process of generating mature translatable mRNAs in eukaryotic gene expression (Baralle & Baralle, 2018). To stimulate pre‐mRNAs splicing, a large ribonucleoprotein (RNP) complex known as the spliceosome can recognize numerous splicing signals, which include the conserved splice sites (5′ splice donor site and 3′ splice acceptor site), the branch site, polypyrimidine track, exonic/intronic splicing enhancers (ESEs/ISEs), and silencers (ESSs/ISSs), as well as other regulatory components or the RNA secondary structure (Dufner‐Almeida et al., 2019). Point variants within coding exons, including missense, synonymous, or nonsense variants, can also result in abnormal pre‐mRNA splicing by disrupting splicing signals (Baeza‐Centurion et al., 2020; Cartegni et al., 2002).

We found that in the last large sample study reported in 2015 (Takeuchi et al., 2015), 88 missense variants were only predicted by ESEfinder software as candidate variants that could induce exon skipping in the *SLC12A3* gene. Moreover, most novel variants analyses were performed to predict the impact on mRNA and protein at the genome level and only in a few cases at both DNA and RNA levels (Ko & Kim, 2021; Takeuchi et al., 2015). Herein, we aimed to explore the functional effects of more than 300 previously described *SLC12A3* missense variants that have not been functionally validated on pre‐mRNAs splicing by bioinformatics tools and minigene assays.

## 2 MATERIALS AND METHODS

### 2.1 Variant nomenclature

The nomenclature for the description of variants was based on the guidelines of the Human Genome Variation Society (http://varnomen.hgvs.org). Nucleotide number was grounded in the *SLC12A3* cDNA sequence (NC_000016.10, NM_000339.3), with c.1 denoting the first nucleotide of the translation initiation codon.

### 2.2 Bioinformatics predictions and screening criteria

All *SLC12A3* missense variants were collected from the Human Gene Mutation Database and ClinVar (October 2020). Bioinformatics software was performed to predict the effects of these variants on pre‐mRNAs splicing. Firstly, analysis of BDGP (http://www.fruitfly.org) was carried out to explore the potential effects on consensus 5′ donor or 3′ acceptor sites and/or to predict the generation and/or activation of novel sites. The satisfying exons with BDGP score below 0.7 were selected to continue the analyses. Then, the Human Splicing Finder system (HSF, https://www.genomnis.com/access‐hsf) was used for all missense variants in these exons to assess the impacts upon exonic splicing regulatory elements (ESEs broken and/or new ESSs creation), and variants with HSF score (ESE/ESS motifs ratio) less than −8 were selected for further minigene splicing assays. In addition, *SLC12A3* variants, which located within 2 bases at the 5′ or 3′ end of the exon and significantly reduce BDGP score, were also selected as candidates for experimental analyses in this study.

### 2.3 Minigene constructions and site‐directed mutagenesis

With informed consent of the subjects and the approval from the Ethics Committee of Qingdao Municipal Hospital affiliated with Qingdao University, genomic DNA was obtained from blood samples of healthy individuals using GenElute Blood Genomic DNA Extraction Kit (Sigma, NA2010). In vitro splicing assay, the fragment consisting of a target exon and flanking introns with 50–200 nucleotides is amplified by specific primers (Table S1), which contain *Xho*I and *Nhe*I restriction sites (*Xho*I: CCGC^CTCGAG; *Nhe*I: CTAG^CTAGC) and were designed by PP5 and Primer‐Blast (http://www.ncbi.nlm.nih.gov/tools/primer‐blast). Moreover, the truncated introns at both ends have no activation of cryptic splicing as verified by HSF. Then, PCR products purified by Gel Extraction Kit (Cwbio, China) and pSPL3 exon trapping vector were, respectively, digested by enzymes *Xho*I and *Nhe*I. PCR products were cloned into the exon trapping vector and plasmid DNA was extracted from individual clones using PurePlasmid Mini Kit (Cwbio, China). The wild‐type (WT) minigene was finally constructed after sequencing using forward and reverse primers and sequence analysis and alignment using Chromas 2.6.5 and Vector NTI Advance 11.

With the extension of the mutagenesis primers (Table S2) and PCR amplification, variants of interest were effectively introduced into the WT plasmids by the GeneArt™ Site‐Directed Mutagenesis PLUS System (Thermo Fisher Scientific, Massachusetts, USA) according to the manufacturer's instruction. The specific reaction system is the same as previously reported (Wang et al., 2020; Zhang et al., 2021). All constructed minigenes were further confirmed by direct sequencing.

### 2.4 Minigene splicing assay

Human epithelial kidney 293 T (HEK 293 T) cells and Hela cells were, respectively, cultured in flasks containing DMEM medium, 10% fetal bovine serum (FBS), penicillin (100 U/L) and streptomycin (100 mg/L), and incubated in a 5% CO_2_ incubator at 37°C. Cells need to be transferred to 12‐well culture plates with an antibiotic‐free medium, and after growing to about 70–80%, the transfection of each group (empty pSPL3‐control (EV), pSPL3‐WT, and pSPL3‐Mutation) was performed with OPTI‐MEM® IMedium and Lipofectamine 2000 (Invitrogen, Carlsbad, CA, USA) following the instruction. Forty‐eight hours later, TRIzol reagent (Invitrogen, USA) was used to extract total RNA, which was produced into cDNA by RT‐PCR (reverse transcription PCR) using Superscript II Reverse Transcriptase (Invitrogen Corporation, Carlsbad, CA) under the instruction booklet of manufacturer.

In order to assess the pattern of transcripts, the PCR amplification reaction was carried out with the vector‐specific primers (SD6 (the forward primer: 5′‐TCTGAGTCACCTGGACAACC‐3′) and SA2 (the reverse primer: 5′‐ATCTCAGTGGTATTTGTGAGC‐3′)), as previously described (Wang et al., 2020; Zhang et al., 2021). Finally, PCR products were identified by 1.5% agarose gel electrophoresis, and each band was accurately quantified by Image J software. The percentage of exon exclusion (%) = (lower band/[lower band + upper band]) × 100. Error bars represent SEM (*n* = 3). *p < .05, independent‐samples T test by GraphPad Prism (Version 6.02, GraphPad Software, USA). Besides, the purification of target DNA bands was performed with a Gel Extraction Kit (CWBIO, China), followed by the sequencing and analysis of all transcripts. Only when the difference between splicing pattern and the WT minigene was observed in both cell lines was it considered significant that the variation resulted in a splicing defect.

## 3 RESULTS

A total of 342 missense variants, distributed in 26 exons of *SLC12A3*, were collected for bioinformatics predictions. They were analyzed in silico with BDGP for splice site prediction, and with ESE/ESS estimation algorithms integrated into HSF. It is well‐known that regulatory elements are common in exons with weak splice sites (Wu et al., 2005). Therefore, we sought to select variants located in exons that have a weak 5′ or 3′ splice site. Except for variants located in the first and last exon that could not be analyzed with the minigene approach, four potential splicing variants were selected following the screening criteria (BDGP and HSF score below 0.7 and − 8, respectively). We also selected six variants within two bases of 5′ or 3′ ends of the exons, which were predicted to have a big influence on classic splicing sites according to BDGP. As a result, a total of 10 candidate variants (c.602G>A, c.602G>T, c.1452C>G, c.1567G>A, c.1667C>T, c.1925G>A, c.2548G>C, c.2549G>C, c.2755A>T, and c.2863A>T) located in seven exons of the *SLC12A3* gene were included in this study and collected from literatures (Balavoine et al., 2011; Colussi et al., 2007; Fujimura et al., 2019; Jiang et al., 2015; Miao et al., 2009; Riveira‐Munoz et al., 2007; Vargas‐Poussou et al., 2011). These candidate variants involved the generation of ESSs and/or the destruction of ESEs, the activation of a cryptic splice site, or the alteration of canonical splice sites (Table 1).

**TABLE 1:** *SLC12A3* exonic variants selected from this study and the results of in silico analyses

Variant		Exon	Exon length (bp)	Location in exon (bp)^a^	BDGP	HSF
c.602G>A	p.Gly201Asp	5	140	1	3'AS: 0.83 → 0.57 (31.33%)^b^	NA
c.602G>T	p.Gly201Val	5	140	1	3'AS: 0.83 → 0.35 (57.83%)^b^	NA
c.1452C>G	p.Cys484Trp	12	124	9	NA	−9^c^
c.1567G>A	p.Ala523Thr	12	124	−1	5'DS: 1.00 → 0.45 (55%)^b^	NA
c.1667C>T	p.Pro556Leu	13	102	−3	NA	−9^c^
c.1925G>A	p.Arg642His	15	100	−1	5'DS: 0.99 → 0.68 (31.31%)^b^	Broken WT DS
c.2548G>C	p.Gly850Arg	21	102	−1	5'DS: 0.85 → 0.22 (74.12%)^b^	Broken WT DS
c.2549G>C	p.Gly850Ala	22	112	1	3'AS: 0.52 → NA	NA
c.2755A>T	p.Arg919Trp	24	136	8	NA	−8^c^
c.2863A>T	p.Ile955Phe	24	136	−21	NA	−10^c^

*Note*: SLC12A3 reference sequence: NC_000016.10, NM_000339.3.

Abbreviations: AS, acceptor splice sites; DS, donor splice sites; WT, wild‐type; NA, not applicable.

^a^ Location of 10 variants relative to the nearest splice site; “+” indicates close to 3'AS; “−” indicates close to 5'DS.

^b^ Score changes with BDGP expressed in percentage.

^c^ ESE/ESS motifs ratio (ESE, exonic splicing enhancer; ESS, exonic splicing silencer).

**FIGURE 1:**
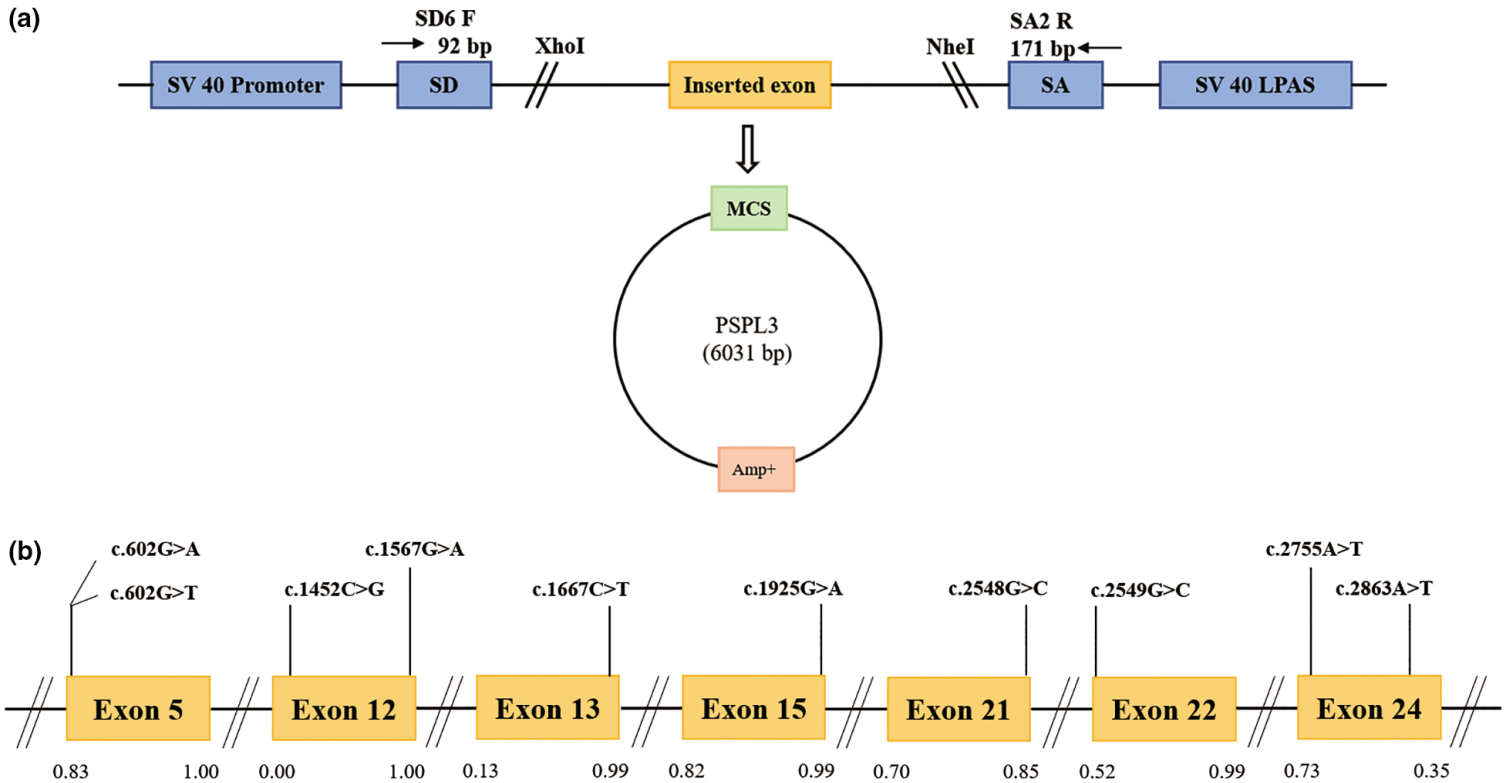
Schematic representation of the minigene splicing assay constructed by pSPL3 vector and position of presumed missense variants we studied in SLC12A3 gene. Panel (a): Transcription begins at the SV40 promoter and ends at the LPAS (late poly(A) signal). The pSPL3 vector contains two exons (SD and SA) and a functional intron. The target exon with partial flanking introns was inserted into pSPL3 vector via XhoI and NheI cloning sites to form the wild‐type or mutant plasmid. MCS, multiple cloning sites. Panel (b): Position of 10 variants. Orange boxes and black lines between them, respectively, indicate the coding exons and introns sequences and their sizes are out of proportion. The BDGP scores of donor and acceptor splice sites are represented in decimal, as shown at the bottom.

**FIGURE 2:**
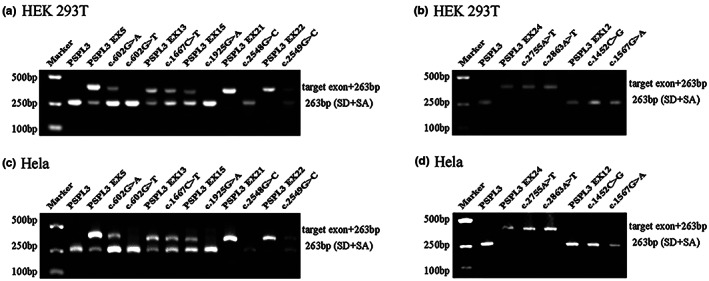
Agarose gel electrophoresis of RT‐PCR products expressed from the SLC12A3 minigenes in HEK 293T and Hela cells, respectively. (a, c) Gel electrophoresis with desired results in HEK 293T and Hela cells, respectively. Lane 1: Marker; lane 2: Empty vector pSPL3 (263 bp); line 3: pSPL3 Ex5 (403 bp (140 bp + 263 bp) and 263 bp); line 4: c.602G>A (403 bp and 263 bp); line 5: c.602G>T (263 bp); line 6: pSPL3 Ex13 (365 bp (102 bp + 263 bp) and 263 bp); line 7: c.1667C>T (365 bp and 263 bp); line 8: pSPL3 Ex15 (363 bp (100 bp + 263 bp) and 263 bp); line 9: c.1925G>A (263 bp); line 10: pSPL3 Ex21 (365 bp (102 bp + 263 bp)); line 11: c.2548G>C (263 bp); line 12: pSPL3 Ex22 (375 bp (112 bp + 263 bp)); line 13: c.2549G>C (375 bp and 263 bp). (b, d) Gel electrophoresis with unpredicted results in HEK 293T and Hela cells, respectively. Lane 1: Marker; lane 2: Empty vector pSPL3 (263 bp); line 3: pSPL3 Ex24 (387 bp (124 bp + 263 bp)); line 4: c.2755A>T (387 bp); line 5: c.2863A>T (387 bp); line 6: pSPL3 Ex12 (263 bp); line 7: c.1452C>G (263 bp); line 8: c.1567G>A (263 bp).

Based on this bioinformatics data, we performed minigene splicing assays in vitro. Taking the corresponding WT minigenes of each exon (pSPL3 Ex5, pSPL3 Ex12, pSPL3 Ex13, pSPL3 Ex15, pSPL3 Ex21, pSPL3 Ex22, and pSPL3 Ex24) as the template, we successfully constructed all candidate variants minigenes through site‐directed mutagenesis (Figure 1). Results of minigene analysis indicated that 60% (6/10) of them effectively disturbed the normal pre‐mRNA splicing as expected (Figure 2a,c), and these were further confirmed by sequencing analysis (Figure 4). Among ten candidates selected by BDGP and HSF, eight variants (c.602G>A, c.602G>T, c.1452C>G, c.1567G>A, c.1667C>T, c.1925G>A, c.2548G>C, and c.2549G>C) led to exon skipping and two variants (c.2755A>T and c.2863A>T) caused no exon skipping. Although two variants (c.1452C>G and c.1567G>A) resulted in exon 12 skipping, the WT minigene of exon 12 (pSPL3 Ex12) also displayed complete exon skipping, making results for these two variants uninterpretable. On the basis of the results, the pathogenicity of these variants was revaluated and classified according to the guidelines of ACMG (Table S4).

### 3.1 As expected, presumed missense variants c.602G>A (p.Gly201Asp) and c.602G>T (p.Gly201Val) induced skipping of exon 5 compared with the WT plasmids

**FIGURE 3:**
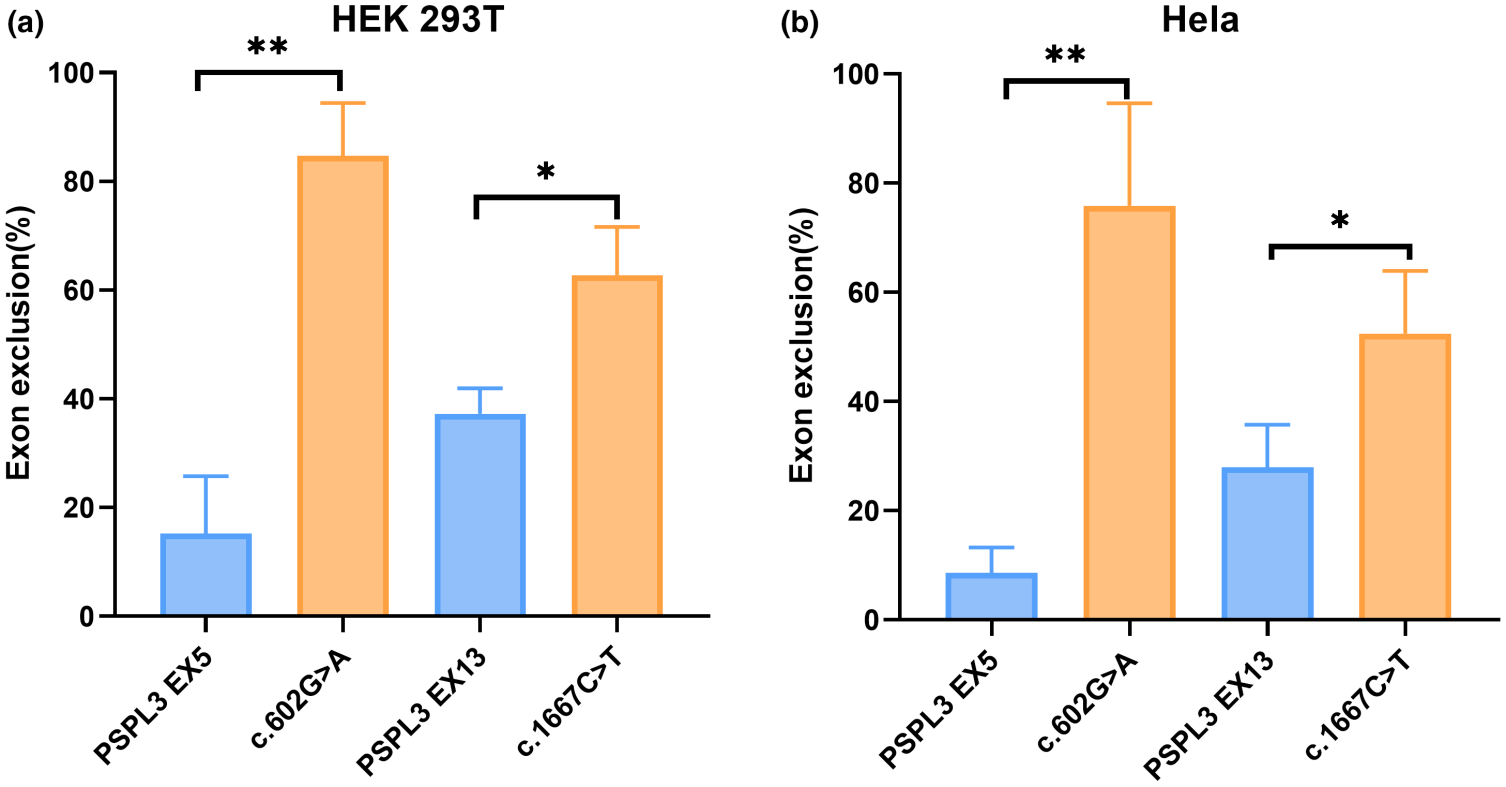
Statistical analysis of RT‐PCR products in HEK 293T (a) and Hela cells (b).The percentage of exon exclusion (%) = (lower band/[lower band + upper band]) × 100. Error bars represent SEM (*n* = 3). *p < .05; **p < .01, independent‐samples *T* test.

**FIGURE 4:**
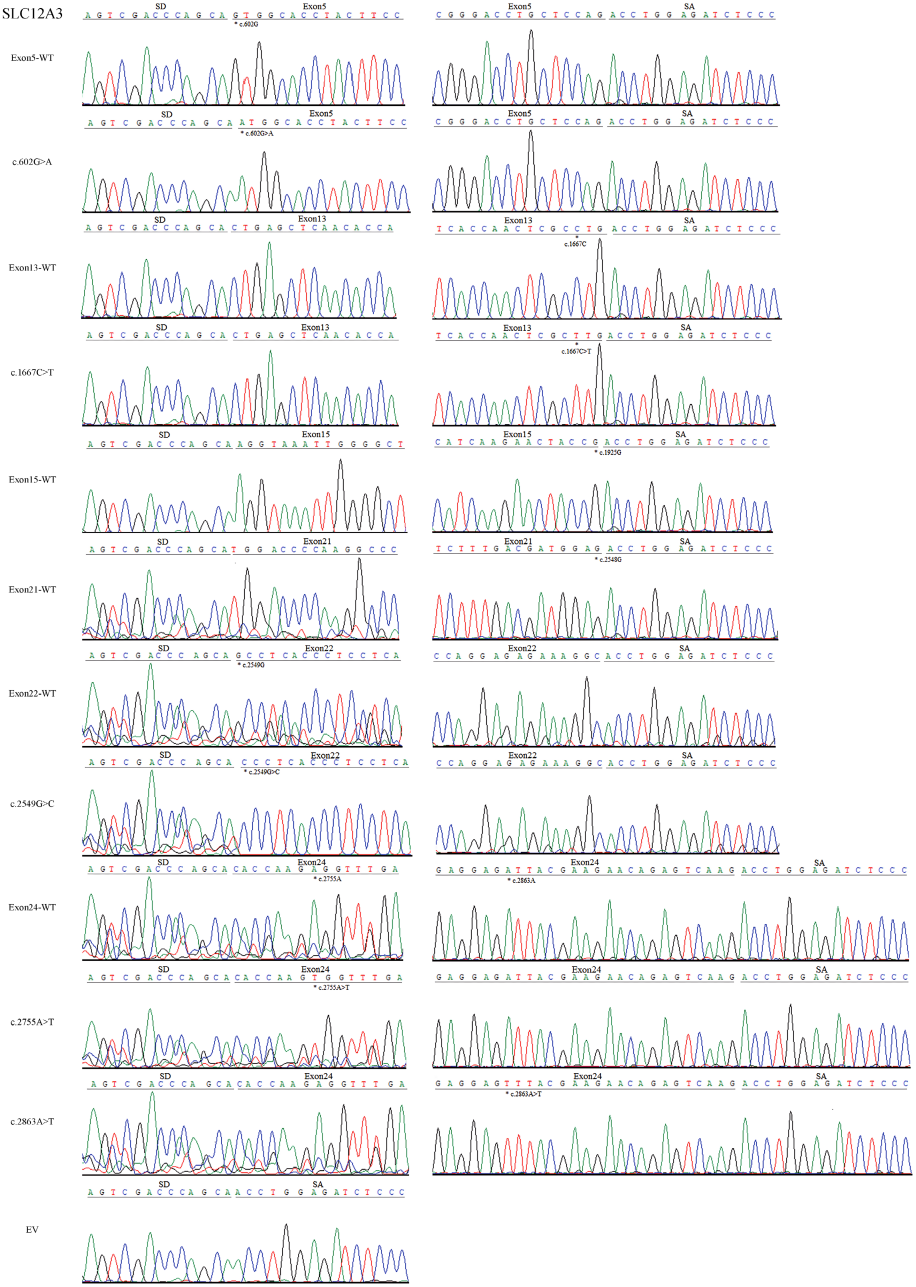
Sanger sequencing figure: Direct sequencing results showed that the larger fragment of each lane in Figure 2 contained the corresponding exon, flanked by two exons of the pSPL3 vector (SD and SA), while the smaller one included only the 3′ and 5′ pSPL3 exons (EV); “*” indicates the mutation site.

Missense variants c.602G>A (p.Gly201Asp) and c.602G>T (p.Gly201Val) both affected the G at the first nucleotide in exon 5 of *SLC12A3*. Bioinformatics predictions from BDGP showed that the variants reduced the score of the WT 3'acceptor splice site from 0.83 to 0.57 and 0.35, respectively (Table 1). To observe the effects of the two variants on pre‐mRNA splicing, the WT (pSPL3 Ex5) and mutants (c.602G>A and c.602G>T) minigenes were transfected individually into HEK 293 T and Hela cells. The result of the minigene assays revealed that two different electrophoresis bands were detected in the WT and the mutant (c.602G>A) lane: a larger band of 403 bp and a smaller band of 263 bp. Meanwhile, the mutant lane of c.602G>T revealed one unique band of 263 bp in line with skipping of exon 5 (Figure 2a,c). Sanger sequencing results showed that the larger fragment contained SLC12A3 exon 5 flanked by two exons of the pSPL3 vector (SD and SA), while the smaller one included only the 3′ and 5′ pSPL3 exons (Figure 4). Quantitative analysis of cDNA products obtained from HEK 293 T and Hela cells indicated that there was an increase of the exon 5‐skipping transcript of c.602G>A corresponded to the WT plasmids (84.65% versus 15.24% in HEK 293 T, *p* < .01; and 75.85% versus 8.56% in Hela cells, *p* < .01; Figure 3).

### 3.2 Variant c.1667C>T (p.Pro556Leu) gave rise to skipping of exon 13 as expected

Variant c.1667C>T (p.Pro556Leu), located at the third‐to‐last nucleotide position of exon 13, was predicted by HSF to make a significant alteration of ESE/ESS motifs ratio (−9), broking five ESEs (CGC**C**TG, CGC**C**TGG (ESE_ASF), CGC**C**TGG (ESE_ASFB), GC**C**TGG, C**C**TGGT) and creating four ESSs (GC**T**TGG, GC**T**TGGT, C**T**TGGT, **T**TGGTA) (Table 1, Table S3). In addition, the splicing effect of exon 13 is affected by a weak acceptor site, and the BDGP score of this site is 0.13 (Table 1). As a result of minigene splicing assays, the WT lane showed two different electrophoresis bands, representing a mature mRNA and a defected mRNA without exon 13 (37.26% in HEK 293T and 27.91% in Hela cells). Mutant (c.1667C>T) also generated two different transcripts, including the larger one with the variant and the shorter one with exon 13 skipping compared with the normal transcript (Figure 2a,c). The results were further confirmed by sequencing analysis (Figure 4). The analysis of the PCR products suggested that the rate of transcript without exon 13 to the full transcripts was 62.76% in HEK 293 T and 52.45% in Hela cells, respectively (*p* < .05, Figure 3).

### 3.3 Variant c.1925G>A (p.Arg642His) led to exon 15 skipping as expected

Variant c.1925G>A (p.Arg642His) caused by the last nucleotide substitution in exon 15, was demonstrated that it reduced the score of the WT donor site from 0.99 to 0.68 with BDGP and also broken the WT donor site with HSF (Table 1, Table S3), most probably affecting splicing. Taken together, we used the WT (pSPL3 Ex15) and control mutant minigene to examine the experimental effect of variant c.1925G>A. The final PCR analysis result revealed that the WT lane, respectively, showed two different fragments of 363 bp and 263 bp, in contrast the mutant lane just showed one unique fragment of 263 bp corresponding to the lack of exon 15 of mRNA (Figure 2a,c). Therefore, variant c.1925G>A (p.Arg642His) disturbed the 5′ DS and led to exon 15 skipping. Sequencing analysis confirmed this variant caused exon 15 skipping (Figure 4).

### 3.4 Variant c.2548G>C (p.Gly850Arg) prevented incorporation of exon 21 into the mature mRNA as expected

Variant c.2548G>C (p.Gly850Arg) was identified at the last nucleotide of exon 21 and was predicted to decrease the score of the WT 5′ DS from 0.85 to 0.22 with BDGP (Table 1). Simultaneously, HSF predicted that Variant c.2548G>C could lead to the deletion of exon 21 by inactivating the splicing donor site (Table S3). Through the WT (pSPL3 Ex21) and mutant (c.2548G>C) minigene analysis, the satisfactory result showed that a short band of 263 bp was detected in the mutant lane and a long band of 365 bp was detected in the wild‐type lane, indicating that the mutant only produced the exon 21‐excluded transcript compared with the original exon 21‐included transcript of the WT (Figure 2a,c). The results were further confirmed by sequencing analysis (Figure 4).

### 3.5 Variant c.2549G>C (p.Gly850Ala) resulted in skipping of exon 22 as expected

Variant c.2549G>C (p.Gly850Ala) affected the G at the first nucleotide in exon 22. Bioinformatic analysis of BDGP revealed that the score of the acceptor site is 0.52, however, it could not be analyzed after the nucleotide substitution (Table 1). The minigene assays result identified that cDNA products generated by the mutant and WT (pSPL3 Ex22) minigenes were different. The lane of WT demonstrated one fragment of 375 bp that contains exon 22, whereas mutant c.2549G>C generated two different fragments of 263 bp and 375 bp, respectively (Figure 2a,c). Because the WT minigene only generated the transcript with exon 22, it was obvious that the mutant (c.2549G>C) resulted in skipping of exon 22, which were confirmed by sequencing analysis (Figure 4).

### 3.6 Not as expected, variants of c.2755A>T (p.Arg919Trp) and c.2863A>T (p.Ile955Phe) in exon 24 did not alter pre‐mRNA splicing

The pre‐mRNA splicing of exon 24 is regulated by a weak donor site, and the BDGP score of this site is 0.35 (Table 1). Variants c.2755A>T, identified at the 8th nucleotide of exon 24, was predicted by HSF to make a significant alteration of ESE/ESS motifs ratio (−8) (Table 1 and Table S3). Variant c.2863A>T was located at the 21st‐to‐last nucleotide position of exon 24 and was predicted to alter splicing with an HSF score of −10 (Table 1 and Table S3). However, analysis of the minigenes demonstrated that both the WT (pSPL3 Ex24) and mutants (c.2755A>T and c.2863A>T) just generate the exon 24‐included transcript (Figure 2b,d), which were confirmed by sequencing analysis (Figure 4), indicating that the two variants did not alter pre‐mRNA splicing.

### 3.7 The WT minigene of exon 12 (pSPL3 Ex12) and mutants (c.1452C>G and c.1567G>A) only produced the exon 12‐excluded transcript beyond the expected

Variant c.1452C>G (p.Cys484Trp), caused by the 9th nucleotide substitution in exon 12, was predicted to disturb pre‐mRNA splicing with an HSF score of −9 (Table 1 and Table S3). Variant c.1567G>A (p.Ala523Thr) affected the G at the last nucleotide of exon 12, and was predicted to decrease the score of the donor site from 1 to 0.45 with BDGP (Table 1). Regrettably, the result of the minigene assays showed that a band of 263 bp (the exon 12‐excluded transcript) was both detected in the WT and the mutant lane (Figure 2b,d). Direct sequencing results showed that all transcripts only contained 3′ and 5′ pSPL3 exons (EV) (Figure 4). Therefore, although these two mutants (c.1452C>G and c.1567G>A) resulted in exon 12 skipping, there was no significant difference from the WT minigene (pSPL3 Ex12).

## 4 DISCUSSION

Alternative splicing, which is present in over 90% of mammalian genes, plays an important role in biodiversity and biocomplexity, and the misregulation of splicing is implicated in an increasing number of human diseases. About 10% of HGMD‐reported human pathogenic variants have been found to influence pre‐mRNA splicing (Soemedi et al., 2017), which is likely an underestimate. Therefore, it is necessary to study the effects of exonic variants in the *SLC12A3* gene of GS on potential splicing, with the discovery of novel variants. In the absence of RNA samples to study alternative splicing, minigene analysis, the effectiveness of which has been confirmed by different studies (Fraile‐Bethencourt et al., 2019; Suarez‐Artiles et al., 2018; Takeuchi et al., 2015; Tournier et al., 2008), is still the most direct and credible experimental approach to assess whether a variant influences recognition of an exon and potentially causes phenotypic changes (Bao et al., 2019). In previous studies, we have used this approach to assess the consequences on pre‐mRNA splicing of presumed mutations associated with different diseases (Han et al., 2019; Wang et al., 2020; Zhang et al., 2021).

Gene splicing diversity is dramatically affected not only by canonical splice signals such as splice sites recognized by the core spliceosomal components but also by a large number of regulatory elements in the exon or flanking intronic sequences. Based on the above, we empirically chose BDGP and HSF as bioinformatics tools to predict the effect of missense variants of *SLC12A3* gene, and obtained some satisfactory results through minigene assays. Variants located at or near to classical splice sites (DS or AS) could result in skipping of the corresponding exon either by generating new splice sites or by inducing a significant reduction of splice site strength, as confirmed by the minigene analysis of variants c.602G>A, c.602G>T, c.1925G>A, c.2548G>C, and c.2549G>C in the study. Additionally, variants in the exon regulated by a weak donor or acceptor splicing site could modify splicing regulatory sequences (disrupt ESEs or create ESSs), leading to the corresponding exon skipping, as revealed by the minigene analysis of variants c.1667C>T in our study. Among the 10 candidate variants selected by BDGP and HSF following the screening criteria, 6 variants causing exon skipping were predicted successfully, which indicated a degree of concordance between in silico predictions and the experimental results. Despite a considerable degree of inaccuracy, it is still valuable to use these two bioinformatics tools to predict the impact of *SLC12A3* exonic variation on splicing based on this or more stringent criteria.

According to the resulting data, among six variants that were predicted successfully, c.602G>T, c.1925G>A, and c.2548G>C were complete splicing mutations just generating the corresponding exon‐excluded transcripts, while the other variants (c.602G>A, c.1667C>T, and c.2549G>C) still produced a little part of transcripts that contained the corresponding variant and were the same size as WT products. Therefore, these three variants probably have a dual damaging effect: on the one hand, a portion of *SLC12A3* transcripts are deleterious due to the exon skipping, which would result in loss of the corresponding proteins, and on the other hand, the remaining mRNA is destructive due to the replacement of a single amino acid, which could lead to the malfunctioning of NCCT. The true splicing effect of these variants needs to be confirmed by analyzing the mRNA from the patient, which we did not have yet.

However, it is worth noting that bioinformatics software and minigene assays, as effective tools for identifying splicing malfunctions, have the limitation in detecting and simulating all splicing patterns compared to the in vivo situation. Variants of c.2755A>T and c.2863A>T, predicted by BDGP and HSF to have a significant impact on ESE/ESS motifs, did not cause exon 24 skipping, and the WT minigene of exon 12 only produced the exon 12‐excluded transcript. Coincidentally, the same results that candidate variants did not alter pre‐mRNA splicing were revealed in other studies (Suarez‐Artiles et al., 2018; Wang et al., 2020; Zhang et al., 2021) and there was a previous study that suggested only 18 of the 20 wild‐type minigene constructs produced the desired wild‐type transcripts in the pSPL3 context, improving the awareness of the limitations of minigene assays and emphasizing the importance of sequence context in regulating splicing (Lin et al., 2021). The reasons for these results may be related to the defects of software, the limited transferability of minigenes, the differential expression of splicing factors in cells, the interference of the mRNA secondary structure, etc. The complete skipping of wild‐type exon 12 might also be associated with the inactivation of splicing sites and the difficulty in recognizing splicing sites. Moreover, it has been reported that the role of splicing regulatory elements could be highly context‐dependent (Goren et al., 2006), but the underlying mechanism has been unclear, and our minigene splicing assays might disrupt this sequence context effect leading to exon 12 skipping.

In addition, we have not yet prepared the expected cDNA constructs based on the prediction of the single amino acid substitution and the corresponding exon deletion, nor have we examined the functional activities and the cell surface expression of these mutant NCCTs, which need further investigation.

In order to verify the pathogenicity of exonic variants of the *SLC12A3* gene in GS, variation analyses at both DNA and RNA levels should be performed to predict the impact on mRNA and protein. Minigene splicing assays have revealed new insights and changed the misinterpretation about the functional consequences of certain variants. It is worth mentioning that exon‐skipping methods to correct variants that disturb normal pre‐mRNA splicing have been effectively assessed in rare diseases (Veltrop & Aartsma‐Rus, 2014). Knowledge of the results of exonic splicing variants may have potential therapeutic significance for GS, further emphasizing the importance of minigene splicing analysis.

## 5 CONCLUSION

In summary, we have carried out a large‐scale analysis of exonic variants in *SLC12A3* related to GS through bioinformatics predictions and minigene assays. The results indicated that six previously presumed missense variants should be classified as splicing variations, which induced the corresponding exon skipping. These variants either break ESEs and generate new ESSs, or interfere with the canonical splice sites, causing significant pre‐mRNA splicing alterations, probably associated with their pathogenicity. This study highlights the important role of assessing the effects of missense variants at the mRNA level in GS, as well as the effectiveness of minigene splicing analysis.

## AUTHOR CONTRIBUTIONS

Xiaomeng Shi, Leping Shao and Hong Wang conceived and designed and performed the experiments. Xiaomeng Shi, Ruixiao Zhang, Zhiying Liu, Wencong Guo and Sai Wang performed the experiments. Xiaomeng Shi, Xuyan Liu, Yanhua Lang and Irene Bottillo contributed to the data analysis. Xiaomeng Shi wrote the manuscript. Bingzi Dong and Leping Shao revised the manuscript. All authors had read and approved the final manuscript.

## FUNDING INFORMATION

This study was funded by the National Natural Science Foundation of China (NO. 82170717).

## CONFLICT OF INTEREST

The authors declare that they have no conflict of interest.

## ETHICS STATEMENT

The study was approved by the ethics committee of the Affiliated Qingdao Municipal Hospital of Qingdao University (No. 2018‐028). Informed consent was obtained from all participants included in this study.

## Supporting information


**Table S1** Primers for PCR amplification of exons in *SLC12A3* gene selected from this study
**TABLE S2** Mutagenesis primers of exonic variants in *SLC12A3* selected from this study
**TABLE S3** The result of the exonic variants predicted by HSF
**TABLE S4** Classification of the variants according to ACMG

## Data Availability

The datasets generated during the current study can be found in the Human Gene Mutation Database (http://www.hgmd.org) and ClinVar (https://www.ncbi.nlm.nih.gov/clinvar/).
