# Supplementary material for: Minigene splicing assays reveal new insights into exonic variants of the SLC12A3 gene in Gitelman syndrome
Source: Mol Genet Genomic Med. 2023 Jan 3;11(4):e2128. doi: 10.1002/mgg3.2128 (PMC10094094; doi:10.1002/mgg3.2128)
Supplement: Supplementary file 1 — Table S1 Primers for PCR amplification of exons in SLC12A3 gene selected from this study TABLE S2 Mutagenesis primers of exonic variants in SLC12A3 selected from this study TABLE S3 The result of the exonic variants predicted by HSF TABLE S4 Classification of the variants according to ACMG [file MGG3-11-e2128-s001.docx]

**Appendix**

**Table S1：Primers for PCR amplification of exons in *SLC12A3* gene selected from this study.**

| **Exon** | **Forward primer (5’-3’)** | **Reverse primer (5’-3’)** | **Product size (bp)** | |
| --- | --- | --- | --- | --- |
| **Exon 5** | CCGCTCGAGTCGTGGGCTCCTAATCCT | CTAGCTAGCGTGAAGTCAGGCAGAGCAG | | 482 |
| **Exon 12** | CCGCTCGAGAATAGAAACAGACACCAGGACC | CTAGCTAGCCGGGGAGATGAGGAGACAGG | | 360 |
| **Exon 13** | CCGCTCGAGAGACACCATCCCTTTGAGC | CTAGCTAGCAGGCTGGTCACAAACTCC | | 555 |
| **Exon 15** | CCGCTCGAGATTACCTCTGTCCCTCCACG | CTAGCTAGCTCTTGTGGTGGCTGGGTG | | 350 |
| **Exon 21** | CCGCTCGAGGGTTCCTGTTCCACCTGC | CTAGCTAGCATATCACCTGCCTGTGCC | | 490 |
| **Exon 22** | CCGCTCGAGGGGGCAGGAACTCACATA | CTAGCTAGCCCTCCCTGTCGTAGCAAA | | 444 |
| **Exon 24** | CCGCTCGAGCAGTGTCCGATGGGTTTC | CTAGCTAGCAACGACTGTGGCTTGAGGA | | 413 |

**Table S2：Mutagenesis primers of exonic variants in *SLC12A3* selected from this study.**

| **Variant** | **Forward primer (5’-3’)** | **Reverse primer (5’-3’)** |
| --- | --- | --- |
| c.602G>A | CTGCCCTGATAG**A**TGGCACCTACTT | AAGTAGGTGCCA**T**CTATCAGGGCAG |
| c.602G>T | CTGCCCTGATAG**T**TGGCACCTACTT | AAGTAGGTGCCA**A**CTATCAGGGCAG |
| c.1452C>G | GCAGTGCCTTTG**G**GAGGACCAGCTG | CAGCTGGTCCTC**C**CAAAGGCACTGC |
| c.1567G>A | TTCATCATCATC**A**GTAAGGCTCTGC | GCAGAGCCTTAC**T**GATGATGATGAA |
| c.1667C>T | TCACCAACTCGC**T**TGGTAAGCAAAC | GTTTGCTTACCA**A**GCGAGTTGGTGA |
| c.1925G>A | TCAAGAACTACC**A**GTGAGCAGAGCT | AGCTCTGCTCAC**T**GGTAGTTCTTGA |
| c.2548G>C | TTTGACGATGGA**C**GTCAGTGACCCC | GGGGTCACTGAC**G**TCCATCGTCAAA |
| c.2549G>C | TCTCCTTGCCAG**C**CCTCACCCTCCT | AGGAGGGTGAGG**G**CTGGCAAGGAGA |
| c.2755A>T | CCCAGCACCAAG**T**GGTTTGAGGACA | TGTCCTCAAACC**A**CTTGGTGCTGGG |
| c.2863A>T | TCAGATGAGGAG**T**TTACGAAGAACA | TGTTCTTCGTAA**A**CTCCTCATCTGA |

Note: Mutated nucleotide in primers is bold.

**Table S3: The result of the exonic variants predicted by HSF.**

| **Variant** | **HSF** |
| --- | --- |
| c.602G>A | NA |
| c.602G>T | NA |
| c.1452C>G | Signal Interpretation  Alteration of auxiliary sequences  Significant alteration of ESE / ESS motifs ratio (-9)  name position sequence  Sironi_motif2 (New ESS Site) chr16:56880133 CTTTG**G**G  IIE (New ESS Site) chr16:56880134 TTTG**G**G  Fas ESS (New ESS Site) chr16:56880134 TTTG**G**G  Sironi_motif2 (New ESS Site) chr16:56880134 TTTG**G**GA  RESCUE ESE (ESE Site Broken) chr16:56880135 TTG**C**GA  IIE (New ESS Site) chr16:56880135 TTG**G**GA  EIE (ESE Site Broken) chr16:56880136 TG**C**GAG  IIE (New ESS Site) chr16:56880136 TG**G**GAG  Fas ESS (New ESS Site) chr16:56880136 TG**G**GAG  IIE (New ESS Site) chr16:56880137 G**G**GAGG  Fas ESS (New ESS Site) chr16:56880137 G**G**GAGG  ESE_ASFB (New ESE Site) chr16:56880137 G**G**GAGGA  RESCUE ESE (New ESE Site) chr16:56880138 **G**GAGGA  PESE (ESE Site Broken) chr16:56880138 **C**GAGGACC  PESE (New ESE Site) chr16:56880138 **G**GAGGACC |
| c.1567G>A | NA |
| c.1667C>T | Signal Interpretation  Alteration of auxiliary sequences  Significant alteration of ESE / ESS motifs ratio (-9)  name position sequence  EIE (ESE Site Broken) chr16:56882492 CGC**C**TG  ESE_ASF (ESE Site Broken) chr16:56882492 CGC**C**TGG  ESE_ASFB (ESE Site Broken) chr16:56882492 CGC**C**TGG  EIE (ESE Site Broken) chr16:56882493 GC**C**TGG  IIE (New ESS Site) chr16:56882493 GC**T**TGG  Sironi_motif2 (New ESS Site) chr16:56882493 GC**T**TGGT  EIE (ESE Site Broken) chr16:56882494 C**C**TGGT  IIE (New ESS Site) chr16:56882494 C**T**TGGT  IIE (New ESS Site) chr16:56882495 **T**TGGTA |
| c.1925G>A | Signal Interpretation  Broken WT Donor Site Alteration of the WT Donor site,  most probably affecting splicing  name position sequences variation  HSF Donor site (matrix GT) chr16:56885362 CC**G**GTGAGC>CC**A**GTGAGC 88.06>77.97 (-11.46%) |
| c.2548G>C | Signal Interpretation  Broken WT Donor Site Alteration of the WT Donor site,  most probably affecting splicing  name position sequences variation  HSF Donor site (matrix GT) chr16:56893052 GA**G**GTCAGT>GA**C**GTCAGT 92.67>81.51 (-12.04%) |
| c.2549G>C | NA |
| c.2755A>T | Signal Interpretation  Alteration of auxiliary sequences  Significant alteration of ESE / ESS motifs ratio (-8)  name position sequence  PESE (ESE Site Broken) chr16:56902373 CACCAAG**A**  EIE (ESE Site Broken) chr16:56902375 CCAAG**A**  ESE_9G8 (ESE Site Broken) chr16:56902376 CAAG**A**G  ESE_Tra2 (ESE Site Broken) chr16:56902377 AAG**A**G  RESCUE ESE (ESE Site Broken) chr16:56902377 AAG**A**GG  ESE_ASF (ESE Site Broken) chr16:56902377 AAG**A**GGT  IIE (New ESS Site) chr16:56902378 AG**T**GGT  ESE_9G8 (ESE Site Broken) chr16:56902379 G**A**GGTT  ESS_hnRNPA1 (ESS Site Broken) chr16:56902379 G**A**GGTT  IIE (New ESS Site) chr16:56902379 G**T**GGTT  Fas ESS (New ESS Site) chr16:56902379 G**T**GGTT  Fas ESS (ESS Site Broken) chr16:56902380 **A**GGTTT  Signal Interpretation  New Donor splice site Activation of a cryptic Donor site.  Potential alteration of splicing  name position sequences variation  HSF Donor site (matrix GT) chr16:56902376 CAAG**A**GGTT>CAAG**T**GGTT 43.03>70.17 (63.07%) |
| c.2863A>T | Signal Interpretation  Alteration of auxiliary sequences  Significant alteration of ESE / ESS motifs ratio (-10)  name position sequence  PESE (New ESE Site) chr16:56902481 TGAGGAG**T**  PESE (ESE Site Broken) chr16:56902482 GAGGAG**A**T  RESCUE ESE (ESE Site Broken) chr16:56902483 AGGAG**A**  EIE (ESE Site Broken) chr16:56902483 AGGAG**A**  ESE_9G8 (ESE Site Broken) chr16:56902484 GGAG**A**T  RESCUE ESE (ESE Site Broken) chr16:56902484 GGAG**A**T  EIE (ESE Site Broken) chr16:56902484 GGAG**A**T  IIE (New ESS Site) chr16:56902484 GGAG**T**T  EIE (ESE Site Broken) chr16:56902485 GAG**A**TT  EIE (ESE Site Broken) chr16:56902486 AG**A**TTA  Fas ESS (ESS Site Broken) chr16:56902486 AG**A**TTA  Fas ESS (New ESS Site) chr16:56902486 AG**T**TTA  ESE_9G8 (ESE Site Broken) chr16:56902487 G**A**TTAC  EIE (ESE Site Broken) chr16:56902488 **A**TTACG |

Note: the affected nucleotide is bold; NA, not available.

**Table S4: Classification of the variants according to ACMG.**

| **Nucleotide**  **mutation** | **Protein alteration** | **Exon** | **ExAc** | **1000g** | **Mutation Taster** | **PANTHER** | **PolyPhen-2** | **Classification** | **ACMG Evidence** |
| --- | --- | --- | --- | --- | --- | --- | --- | --- | --- |
| c.602G>A | p.Gly201Asp | 5 | 0 | 0 | Disease causing | Probably damaging | Probably damaging | Pathogenic | PVS1+ PM1+PM2+PP3+PP5 |
| c.602G>T | p.Gly201Val | 5 | 0 | 0 | Disease causing | Probably damaging | Probably damaging | Pathogenic | PVS1+ PM1+ PM2+PP3+PP5 |
| c.1452C>G | p.Cys484Trp | 12 | 0 | 0 | Disease causing | Probably damaging | Probably damaging | Likely Pathogenic | PM1+PM2+PP3+PP5 |
| c.1567G>A | p.Ala523Thr | 12 | 1 | 0 | Disease causing | Probably damaging | Probably damaging | Likely Pathogenic | PM1+PM2+PP3+PP5 |
| c.1667C>T | p.Pro556Leu | 13 | 0 | 0 | Disease causing | Probably damaging | Possibly damaging | Pathogenic | PVS1+ PM2+PP3 |
| c.1925G>A | p.Arg642His | 15 | 0 | 0 | Disease causing | Probably damaging | Probably damaging | Pathogenic | PVS1+ PM1+PM2+PP3+PP5 |
| c.2548G>C | p.Gly850Arg | 21 | 0 | 0 | Disease causing | Probably damaging | Probably damaging | Pathogenic | PVS1+ PM2+PP3+PP5 |
| c.2549G>C | p.Gly850Ala | 22 | 0 | 0 | Disease causing | Probably damaging | Probably damaging | Pathogenic | PVS1+ PS3+PS4+PM2+PM3+PP3+PP5 |
| c.2755A>T | p.Arg919Trp | 24 | 0 | 0 | Disease causing | Possibly damaging | Probably damaging | Uncertain Significance | PM2+PP3+PP4 |
| c.2863A>T | p.Ile955Phe | 24 | 0 | 0 | Disease causing | Possibly damaging | Benign | Uncertain Significance | PM2+PP3+PP4 |

Note: PVS, pathogenic very strong; PS, pathogenic strong; PM, pathogenic moderate; PP, pathogenic supporting.
